# Retraction: Modeling Brain Somatic Mosaicism With Cerebral Organoids, Including a Note on Mutant Microglia

**DOI:** 10.3389/fnmol.2020.628401

**Published:** 2020-12-14

**Authors:** Frontiers Editorial Office

The journal retracts the 14 November 2019 article cited above.

Following publication, it was brought to our attention that the author used and published primary research data, which was generated together with other researchers in the laboratory, without permission and without a mention to the other contributors. In particular, the human iPSCs data included does not have permission from the authorities or the patients. Unauthorized data use is a breach of Frontiers guidelines and those of the Committee on Publication Ethics. As such, this article is being retracted.

The author does not concur with the retraction and sincerely regret any inconvenience this may have caused to the reviewers, editors, and readers of Frontiers in Molecular Neuroscience.

